# Microbiology testing around the time of antibiotic initiation among residents of long-term care facilities

**DOI:** 10.1093/jac/dkag212

**Published:** 2026-06-29

**Authors:** Georgina A Hughes, Robert N Jorissen, Yohanes A Wondimkun, Maria C Inacio, Catherine Lang, Noleen Bennett, Leon J Worth, Karin Thursky, Malcolm Clark, Rodney James, Janet K Sluggett

**Affiliations:** School of Allied Health and Human Performance, College of Health, Adelaide University, Adelaide, South Australia, Australia; Registry of Senior Australians Research Centre, South Australian Health and Medical Research Institute, Adelaide, South Australia, Australia; Registry of Senior Australians Research Centre, South Australian Health and Medical Research Institute, Adelaide, South Australia, Australia; Registry of Senior Australians Research Centre, Caring Futures Institute, College of Nursing and Health Sciences, Flinders University, Bedford Park, South Australia, Australia; School of Allied Health and Human Performance, College of Health, Adelaide University, Adelaide, South Australia, Australia; Registry of Senior Australians Research Centre, South Australian Health and Medical Research Institute, Adelaide, South Australia, Australia; Registry of Senior Australians Research Centre, South Australian Health and Medical Research Institute, Adelaide, South Australia, Australia; Registry of Senior Australians Research Centre, Caring Futures Institute, College of Nursing and Health Sciences, Flinders University, Bedford Park, South Australia, Australia; Registry of Senior Australians Research Centre, South Australian Health and Medical Research Institute, Adelaide, South Australia, Australia; Victorian Healthcare Associated Infection Surveillance System (VICNISS) Coordinating Centre, Melbourne Health, Melbourne, Victoria, Australia; National Centre for Antimicrobial Stewardship, Department of Infectious Diseases, The University of Melbourne, Parkville, Victoria, Australia; Department of Nursing, Melbourne School of Health Sciences, The University of Melbourne, Parkville, Victoria, Australia; Victorian Healthcare Associated Infection Surveillance System (VICNISS) Coordinating Centre, Melbourne Health, Melbourne, Victoria, Australia; Sir Peter MacCallum Department of Oncology, The University of Melbourne, Parkville, Victoria, Australia; Department of Medicine, The University of Melbourne, Parkville, Victoria, Australia; National Centre for Antimicrobial Stewardship, Department of Infectious Diseases, The University of Melbourne, Parkville, Victoria, Australia; Sir Peter MacCallum Department of Oncology, The University of Melbourne, Parkville, Victoria, Australia; The Royal Melbourne Hospital Guidance Group, Melbourne Health, Melbourne, Victoria, Australia; Department of General Practice, University of Melbourne, Parkville, Victoria, Australia; National Centre for Antimicrobial Stewardship, Department of Infectious Diseases, The University of Melbourne, Parkville, Victoria, Australia; The Royal Melbourne Hospital Guidance Group, Melbourne Health, Melbourne, Victoria, Australia; School of Allied Health and Human Performance, College of Health, Adelaide University, Adelaide, South Australia, Australia; South Australian Health and Medical Research Institute, Adelaide, South Australia, Australia

## Abstract

**Background and objectives:**

While microbiology tests can guide management of infectious diseases, little is known about the prevalence of testing around the time of antibiotic initiation in long-term care facilities (LTCFs). The objectives of this study were to investigate prevalence and factors associated with microbiology testing around the time of antibiotic initiation, and subsequent treatment pathways in LTCFs.

**Methods:**

This retrospective cohort study included individuals aged ≥65 years who entered a LTCF in three Australian states between 1 January 2017 and 30 June 2019, and received a systemic antibiotic (*n* = 36 977). Prevalence of microbiology testing in the 14 days pre- and 7 days post-antibiotic initiation, and treatment pathways 14 days post-initiation, were determined. Multivariable logistic regression determined adjusted odds ratios (aORs) and 95% confidence intervals (95%CIs) for factors associated with testing.

**Results:**

In total 15 407 (41.7%) individuals were tested around the time of antibiotic initiation, ranging from 22.9% (*n* = 585/2551 residents) of macrolide initiators to 79.3% (*n* = 413/521) of nitrofurantoin initiators. Individuals with urinary tract infections on LTCF entry (aOR 1.24, 95%CI 1.10–1.40) or initiating trimethoprim (aOR 2.80, 95%CI 2.50–3.13) were more likely to be tested. Males (aOR 0.81, 95%CI 0.77–0.85), and residents who received cephalosporins (aOR 0.75, 95%CI 0.68–0.84), penicillins (aOR 0.49, 95%CI 0.45–0.55), or with airways disease (aOR 0.87, 95%CI 0.82–0.91) had lower odds of testing. Among those tested, 14.5% (*n* = 2238) had a second dispensing of the same antibiotic, 11.4% (*n* = 1 751) switched antibiotic therapy and 5.9% (*n* = 904) were hospitalized within 14 days.

**Conclusions:**

With four in ten residents tested, and lower prevalence within certain resident subgroups, this study suggests a high dependence on initiating empiric therapy in LTCFs.

## Introduction

Older people living in long-term care facilities (LTCFs, also known as residential aged care facilities, care homes or nursing homes) are at high risk of infection. Up to 18.7% of residents experience infection signs and symptoms on a single day (pooled point-prevalence of 3.5% across 31 studies), with urinary tract, respiratory and skin and soft tissue infections being the most common.^1^ The communal living environment, frequent care transitions, use of medical devices (e.g. indwelling urinary catheters, feeding tubes), older age and high prevalence of cognitive decline and multimorbidity among residents of LTCFs contribute to higher infection rates, and create diagnostic challenges.^2–4^

Globally, on any given day, ∼5% of residents of LTCFs receive an antibiotic, with 45%–79% of residents treated annually and only 28.5% of antibiotic use considered appropriate.^5,6^ There are widespread concerns about antimicrobial resistance in LTCFs. Approximately one-third of residents have ≥1 multi-drug resistant organism on entry, a further 40% acquire one during their stay,^7^ and outbreaks of multi-drug resistant pneumonia in a Dutch LTCF cost ∼€250 000 to control.^8–10^ This has led to an increased focus on LTCF infection prevention and control (IPC) initiatives globally, including point-prevalence surveillance programmes such as the healthcare-associated infections and antimicrobial use in European LTCFs (HALT) project and the Aged Care National Antimicrobial Prescribing Survey (Aged Care NAPS) in Australia.^4,11,12^

Antimicrobial stewardship initiatives, a key IPC component, outline the need for timely and appropriate microbiological testing to guide prescribing. Microbiological testing enables confirmation of infection and supports the prescriber to administer targeted antibiotics for a specific indication and treatment course. Without testing, the diagnosis may be unknown or uncertain, and antibiotic prescribing is necessarily broad-spectrum and empirical. When used appropriately, microbiology testing can reduce misdiagnosis and optimize treatment efficiency and health outcomes,^13–15^ for example by guiding selection of an effective antibiotic that resolves the infection and avoids hospitalization. In 2023–2024, the HALT study found only 20% of infections had confirmed positive microbiology tests,^11^ and an earlier HALT study reported that one-quarter of prescribed antimicrobials had a related test, with 59% identifying a microorganism.^16^ The collection of both positive and negative microbiological cultures in US LTCFs decreased between 2010 and 2017.^17^ In Australia, microbiology testing around the time of antibiotic initiation was more common in LTCFs than in primary care.^18^ Yet, only one-quarter of residents dispensed an antibiotic with high potential for resistance (3453 of 7388 residents) were tested in the LTCF.^18^ Despite high antibiotic use,^5,6,19^ very few population-level studies have examined the use and factors associated with microbiology testing around the time of antibiotic initiation in LTCFs, or if testing influences antibiotic selection and ongoing treatment pathways.

## Objectives

The objectives of this study were to (i) investigate the prevalence and factors associated with microbiology testing around the time of antibiotic initiation in LTCFs and (ii) determine subsequent treatment pathways within 2 weeks of antibiotic initiation among residents who received a microbiology test and those not tested.

## Methods

### Study design and data source

A retrospective cohort study using the Registry of Senior Australians (ROSA) National Historical Cohort was conducted.^20^ ROSA integrates linked, de-identified health and aged care service use, medical, pharmaceutical, sociodemographic and mortality information for all individuals aged ≥65 years who were assessed for eligibility or accessed Australian Government-subsidised long-term care (LTC) services from 2002 onwards.^20^ Datasets within ROSA and used in this study included those from the Australian Institute of Health and Welfare (AIHW) National Aged Care Data Clearinghouse, including LTCF service records, the National Death Index, Australian Government Medicare Benefits Schedule (MBS; national medical services subsidy) and Pharmaceutical Benefits Scheme (PBS; medicines subsidised nationally) claims, and four Australian state’s hospitalization and emergency department (ED) data collections, which represent ∼87% of individuals accessing LTC services nationally.^20^

### Study cohort and setting

**Figure 1.**
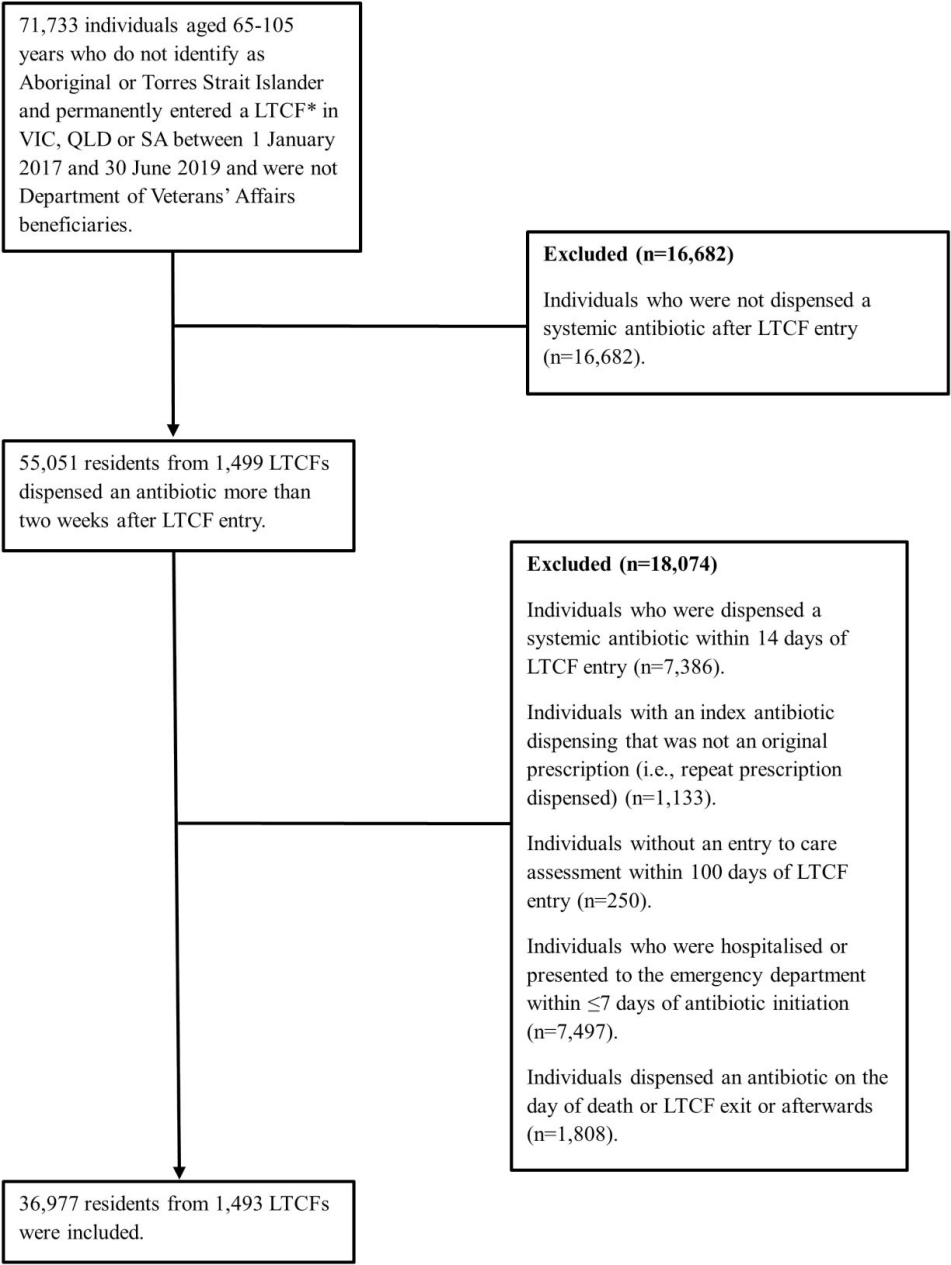
Flow chart for study cohort selection. LTCF, long-term care facility, QLD, Queensland, SA, South Australia, VIC, Victoria. *Individuals who accessed permanent care, or respite care for ≤120 days, within 120 days of entry to permanent care.

Non-Indigenous individuals aged 65–105 years who entered a LTCF in Victoria, Queensland or South Australia between 1 January 2017 and 30 June 2019 and were dispensed a systemic antibiotic at least 2 weeks post-LTCF entry were included. These three Australian states comprise 54% of the national LTCF population.^6^ Analysis of Indigenous individuals’ records requires Indigenous leadership, governance and ethics approval and hence Indigenous people could not be included in this study. Department of Veterans’ Affairs beneficiaries were excluded as access to subsidised medical services (e.g. microbiology testing) may differ compared with non-beneficiaries. Individuals without an entry into care assessment^20^ within 100 days of LTCF entry were also excluded. Date of LTCF entry was the date of permanent entry to a LTCF, or the date of entry to respite care (LTCF-based) where individuals accessed respite immediately before permanent care. To ensure the cohort mostly included LTCF-initiated antibiotics, individuals who presented to the ED and/or were hospitalized in the week before antibiotic initiation were excluded. Individuals with an index antibiotic dispensed from a repeat prescription were also excluded. The final cohort included 36 977 residents (Figure 1).

Residents were screened for initiation of a systemic antibiotic (i.e. first dispensing) at least two weeks post-LTCF entry and were followed for up to 14 days after antibiotic initiation, until death, LTCF exit or 31 December 2019, whichever occurred first (Figure S1, available as Supplementary data at *JAC* Online). World Health Organization (WHO) Anatomical Therapeutic Chemical (ATC) codes (J01*), which are mapped to PBS item codes in the PBS pharmaceutical claims dataset, determined antibiotic use.^19^ Antibiotics were examined overall, by class and by Australia’s classification system for antimicrobial resistance (i.e. ‘access’ antibiotics for low risk first-line treatment, ‘curb’ for high risk first-line treatments and ‘contain’ for high risk antibiotics that are not first-line) (Table S1).^21^ Evidence suggests that Australia’s Priority Antimicrobial List classification system (developed in 2020) is more restrictive than the WHO AWaRe classification (2019), with 40% of antibiotics classified as ‘access’ (i.e. low risk first-line treatments) in Australia versus 70% with WHO AWaRe.^22^ These differences are driven by different classification of cefalexin, amoxicillin/clavulanic acid and cefazolin.^22^

### Outcomes of interest

Outcomes of interest included microbiology testing around the time of antibiotic initiation (i.e. 14 days pre-initiation and 7 days post-initiation). This window was based on a previous study^18^ and visual inspection of a scatter plot of the time between antibiotic initiation and microbiology test processing in our cohort (Figure S2). Microbiology tests (including those identifying bacterial and other pathogens) were ascertained using relevant item codes in MBS groups P3 and P9 (Table S2) and evaluated overall and by type (i.e. urine, skin/superficial, faecal, respiratory, genital, eye/ear/nose/throat, blood, targeted and microbial nucleic acid amplification). Microbiology test use was also explored separately for each antibiotic class to explore differences in prevalence of testing between classes.

Treatment pathways among residents who received a microbiology test and those not tested were determined by sequential examination of the following events in the 14 days post-antibiotic initiation: a subsequent dispensing of (i) the same or (ii) a different antibiotic; (iii) ED presentation (i.e. without hospital admission), (iv) unplanned hospitalization or ED presentation resulting in admission, and (v) LTCF exit/death. Principal diagnoses for ED presentation or hospitalization and primary causes of death were described using International Classification of Diseases Tenth Revision, Australian Modification (ICD-10-AM) codes.

### Covariates

Resident characteristics and medicine-, service- and facility-related factors were used to describe the cohort and were evaluated for inclusion in regression models used to determine factors associated with whether microbiology testing was performed. Sociodemographic factors included age at cohort entry, sex, country of birth (Australia, other), preferred language (English, other), Australian state of residence, LTCF remoteness (major city, outside of major cities) and LTCF ownership type (private, not-for-profit, government).^23,24^ Residents’ health conditions [dementia, diabetes, chronic airways disease, angina, Parkinson’s disease, urinary incontinence, malignancy and history of falls, fractures and urinary tract infections (UTIs)] and required assistance with activities of daily living, behavioural daily living and complex healthcare needs (nil/low, medium and high) were determined from eligibility and entry to LTCF assessments. Pharmaceutical claims in the year before antibiotic initiation were used to ascertain the number of unique medicines dispensed and previous antibiotic use, and claims in the 6 months before LTCF entry determined the Rx-Risk-V Comorbidity Index score.^25^ Service-related factors included the number of hospitalizations and general medical practitioners’ visits (identified from selected MBS claims in groups A01, A02 and A35) in the year before antibiotic initiation. Year of cohort entry, Australian season of antibiotic initiation and days from LTCF entry to antibiotic initiation were also ascertained.

### Statistical analysis

Descriptive statistics summarized resident characteristics, antibiotic initiation and microbiology testing, and treatment pathways during the 14-day follow-up period. Multivariable generalized estimating equation logistic regression models were used to estimate adjusted odds ratios (aORs) and 95% confidence intervals (95%CIs) to evaluate potential factors associated with testing around the time of antibiotic initiation. Factors associated with urine testing (accounting for 72% of tests provided) were also examined. An exchangeable correlation matrix was used to account for LTCF-level clustering. Model assumptions were assessed and met, and Hosmer–Lemeshow tests were used to assess model goodness-of-fit (a priori value >0.05). Complete case analysis was undertaken (*n* = 611 (1.7%) missing data). SAS, version 9.4 (SAS Institute Inc., Cary, NC, USA) was used for all analyses. R statistical package version 4.1.3 and Microsoft Excel were used for graphical presentation.

### Ethical approval

This study was approved by the University of South Australia Human Research Ethics Committee (HREC) (ref. 200489) with ongoing approval and project monitoring transferred to the new, superseding Adelaide University HREC, the AIHW HREC (ref. EO2022/4/1376) and the South Australian Department for Health & Wellbeing HREC (ref. HREC/18/SAH/90) for the inclusion of South Australia, Victoria and Queensland datasets.

## Results

Overall, 36 977 individuals from 1493 LTCFs were included (Table 1). Of these, 23 297 (63.0%) were female, 19 681 (53.2%) were aged ≥85 years on cohort entry and 33 021 (89.3%) spoke English as their primary language. Residents had a median comorbidity score of 5 (IQR 3–7), and 18 330 (49.6%) lived with dementia, 8689 (23.5%) had diabetes and 9742 (26.3%) had airways disease.

**Table 1.** Characteristics of individuals in study cohort

Characteristic	Total cohort (*n* = 36 977) [*n* (%)]	Received a microbiology test (*n* = 15 407) [*n* (%)]	Did not receive a microbiology test (*n* = 21 570) [*n* (%)]
Cohort entry year			
2017	16 231 (43.9)	6 590 (42.8)	9 641 (44.7)
2018	14 778 (40.0)	6 195 (40.2)	8 583 (39.8)
2019	5 968 (16.1)	2 622 (17.0)	3 346 (15.5)
Age on cohort entry (years)			
Median (IQR)	85 (80–89)	85 (80–89)	85 (80–89)
0–74	4 187 (11.3)	1 677 (10.9)	2 510 (11.6)
75–84	13 109 (35.5)	5 404 (35.1)	7 705 (35.7)
85–95	17 848 (48.3)	7 542 (49.0)	10 306 (47.8)
≥90	1 833 (5.0)	784 (5.1)	1 049 (4.9)
Female sex	23 297 (63.0)	10 426 (67.7)	12 871 (59.7)
Born in Australia^a^	24 318 (65.8)	10 206 (66.2)	14 112 (65.4)
Primary language is English^a^	33 021 (89.3)	13 935 (90.4)	19 086 (88.5)
RxRisk-V comorbidity score on LTCF entry^b^			
Median (IQR)	5 (3–7)	5 (3–7)	5 (3–7)
0–3	9 946 (26.9)	4 230 (27.5)	5 716 (26.5)
4–5	10 478 (28.3)	4 398 (28.5)	6 080 (28.2)
6–7	9 360 (25.3)	3 862 (25.1)	5 498 (25.5)
≥8	7 193 (19.5)	2 917 (18.9)	4 276 (19.8)
Health conditions on LTCF entry			
Dementia	18 330 (49.6)	7 905 (51.3)	10 425 (48.3)
Diabetes	8 689 (23.5)	3 665 (23.8)	5 024 (23.3)
Chronic airways disease	9 742 (26.3)	3 659 (23.7)	6 083 (28.2)
Angina	3 665 (9.9)	1 500 (9.7)	2 165 (10.0)
Parkinson’s disease	3 327 (9.0)	1 393 (9.0)	1 934 (9.0)
Urinary incontinence	6 669 (18.0)	2 892 (18.8)	3 777 (17.5)
Malignancy	6 838 (18.5)	2 827 (18.3)	4 011 (18.6)
Urinary tract infection	1 331 (3.6)	701 (4.5)	630 (2.9)
History of falls	7 203 (19.5)	3 005 (19.5)	4 198 (19.5)
History of fracture	2 534 (6.9)	1 106 (7.2)	1 428 (6.6)
Need for assistance with activities of daily living			
None or low	5 830 (15.8)	2 217 (14.4)	3 613 (16.8)
Medium	13 131 (35.5)	5 472 (35.5)	7 659 (35.5)
High	18 016 (48.7)	7 718 (50.1)	10 298 (47.7)
Need for assistance with behavioural daily living			
None or low	8 087 (21.9)	3 395 (22.0)	4 692 (21.8)
Medium	9 892 (26.8)	4 156 (27.0)	5 736 (26.6)
High	18 998 (51.4)	7 856 (51.0)	11 142 (51.7)
Need for assistance with complex health care			
None or low	8 892 (24.0)	3 622 (23.5)	5 270 (24.4)
Medium	13 323 (36.0)	5 498 (35.7)	7 825 (36.3)
High	14 762 (39.9)	6 287 (40.8)	8 475 (39.3)
Number of unique medicines dispensed in the year before antibiotic initiation			
Median (IQR)	11 (8–16)	11 (8–16)	11 (8–16)
0–7	8 623 (23.3)	3 590 (23.3)	5 033 (23.3)
8–11	10 191 (27.6)	4 267 (27.7)	5 924 (27.5)
12–15	8 265 (22.4)	3 433 (22.3)	4 832 (22.4)
≥16	9 898 (26.8)	4 117 (26.7)	5 781 (26.8)
Australian state of residence			
Victoria	18 296 (49.5)	6 666 (43.3)	11 630 (53.9)
Queensland	12 537 (33.9)	5 634 (36.6)	6 903 (32.0)
South Australia	6 144 (16.6)	3 107 (20.2)	3 037 (14.1)
Resided in a major city	26 075 (70.5)	10 810 (70.2)	15 265 (70.8)
LTCF ownership			
Not-for-profit	17 393 (47.0)	7 489 (48.6)	9 904 (45.9)
Private	17 306 (46.8)	6 972 (45.3)	10 334 (47.9)
Government	2 278 (6.2)	946 (6.1)	1 332 (6.2)
Number of GP visits in the year before antibiotic initiation			
Median (IQR)	9 (5–16)	9 (5–16)	9 (4–16)
0–11	22 267 (60.2)	9 149 (59.4)	13 118 (60.8)
12–17	6 979 (18.9)	2 945 (19.1)	4 034 (18.7)
18–25	4 449 (12.0)	1 902 (12.3)	2 547 (11.8)
≥26	3 282 (8.9)	1 411 (9.2)	1 871 (8.7)
Number of hospitalizations in the year before antibiotic initiation			
Median (IQR)	1 (0–2)	1 (0–2)	1 (0–2)
0	10 424 (28.2)	4 174 (27.1)	6 250 (29.0)
1	10 836 (29.3)	4 507 (29.3)	6 329 (29.3)
2	6 838 (18.5)	2 941 (19.1)	3 897 (18.1)
≥3	8 879 (24.0)	3 785 (24.6)	5 094 (23.6)
At least one antibiotic dispensing in the year before antibiotic initiation			
Overall	20 526 (55.5)	8 863 (57.5)	11 663 (54.1)
Cephalosporin	11 789 (31.9)	5 259 (34.1)	6 530 (30.3)
Penicillin	10 151 (27.5)	4 214 (27.4)	5 937 (27.5)
Trimethoprim	5 492 (14.9)	2 808 (18.2)	2 684 (12.4)
Tetracycline	3 262 (8.8)	1 191 (7.7)	2 071 (9.6)
Macrolide	2 716 (7.3)	1 007 (6.5)	1 709 (7.9)
Time from LTCF entry to antibiotic initiation (days)			
15–30	4 347 (11.8)	1 958 (12.7)	2 389 (11.1)
31–90	11 844 (32.0)	5 190 (33.7)	6 654 (30.8)
91–365	16 814 (45.5)	6 754 (43.8)	10 060 (46.6)
≥366	3 972 (10.7)	1 505 (9.8)	2 467 (11.4)
Season during which the antibiotic was initiated			
Summer	7 410 (20.0)	3 113 (20.2)	4 297 (19.9)
Autumn	9 309 (25.2)	3 978 (25.8)	5 331 (24.7)
Winter	10 916 (29.5)	4 426 (28.7)	6 490 (30.1)
Spring	9 342 (25.3)	3 890 (25.2)	5 452 (25.3)

GP, general medical practitioner, IQR, interquartile range, LTCF, long-term care facility.

^a^Missing information: country of birth (*n* = 146, 0.4%), primary language (*n* = 288, 0.8%).

^b^The Rx-Risk-V comorbidity score is determined from pharmaceutical claims in the 6 months before LTCF entry.

The median time from LTCF entry to antibiotic initiation was 107 days (interquartile range (IQR) 53–218). Cephalosporins were the most frequently dispensed antibiotic class (*n* = 13 319 residents, 36.0%), followed by penicillins (*n* = 11 517, 31.1%) and trimethoprim (*n* = 6085, 16.5%) (Table 2, Table S3). ‘Curb’ or ‘contain’ antibiotics were initiated for 21 145 (57.2%) residents (Table S4). Most antibiotics dispensed (*n* = 39 198 of 40 734, 96.2%) were prescribed by general medical practitioners and 9.4% (*n* = 3458 residents) were dispensed >1 antibiotic at treatment initiation.

**Table 2.** Number of individuals who initiated an antibiotic, and provision of microbiology tests around the time of antibiotic initiation, overall and by antibiotic type and type of microbiology test

Residents receiving microbiology tests around the time of antibiotic initiation *n* (%)	Any	Cephalosporin	Penicillin	Trimethoprim	Tetracycline	Macrolide	Nitrofurantoin	Lincosamide	Quinolone	Nitroimidazole
Antibiotic initiation	36 977 (100.0)	13 319 (36.0)	11 517 (31.1)	6085 (16.5)	2578 (7.0)	2551 (6.9)	521 (1.4)	471 (1.3)	356 (1.0)	345 (0.9)
Any microbiology test	15 407 (41.7)	5509 (41.4)	3535 (30.7)	4459 (73.3)	656 (25.4)	585 (22.9)	413 (79.3)	167 (35.5)	210 (59.0)	133 (38.6)
Type of microbiology test										
Urine	11 162 (30.2)	4041 (30.3)	1902 (16.5)	4298 (70.6)	220 (8.5)	199 (7.8)	402 (77.2)	47 (10.0)	133 (37.4)	37 (10.7)
Skin/superficial	1983 (5.4)	998 (7.5)	522 (4.5)	119 (2.0)	95 (3.7)	73 (2.9)	8 (1.5)	117 (24.8)	61 (17.1)	18 (5.2)
Respiratory	393 (1.1)	48 (0.4)	199 (1.7)	17 (0.3)	82 (3.2)	60 (2.4)	n/a	n/a	13 (3.7)	n/a
Faecal	322 (0.9)	88 (0.7)	97 (0.8)	56 (0.9)	14 (0.5)	10 (0.4)	n/a	n/a	n/a	63 (18.3)
Genital	140 (0.4)	45 (0.3)	28 (0.2)	30 (0.5)	7 (0.3)	n/a	7 (1.3)	n/a	n/a	25 (7.2)
Eye/ear/nose/ throat	193 (0.5)	57 (0.4)	86 (0.7)	9 (0.1)	18 (0.7)	14 (0.5)	n/a	n/a	n/a	n/a
Blood	178 (0.5)	58 (0.4)	74 (0.6)	23 (0.4)	15 (0.6)	16 (0.6)	n/a	0 (0.0)	n/a	0 (0.0)
Targeted^a^	310 (0.8)	95 (0.7)	91 (0.8)	41 (0.7)	19 (0.7)	13 (0.5)	8 (1.5)	n/a	7 (2.0)	40 (11.6)
Microbial nucleic acid amplification^b^	2587 (7.0)	670 (5.0)	1127 (9.8)	165 (2.7)	310 (12.0)	296 (11.6)	18 (3.5)	23 (4.9)	20 (5.6)	57 (16.5)

n/a, not reported (due to low cell counts). All antibiotic types were counted for *n* = 3458 (9.4%) individuals who initiated >1 type of antibiotic on the same date.

Aminoglycosides, glycopeptides and other systemic antibiotics are not shown due to low prevalence of use (0.2% of antibiotics dispensed) (Table S3).

^a^Targeted tests include Mycobacteria, *Chlamydia trachomatis*, Cryptosporidium, Giardia*, Clostridioides difficile*, Hepatitis virus and Epstein–Barr virus.

^b^Microbial nucleic acid amplification also include some tests that are not specific to other test types.

**Figure 2.**
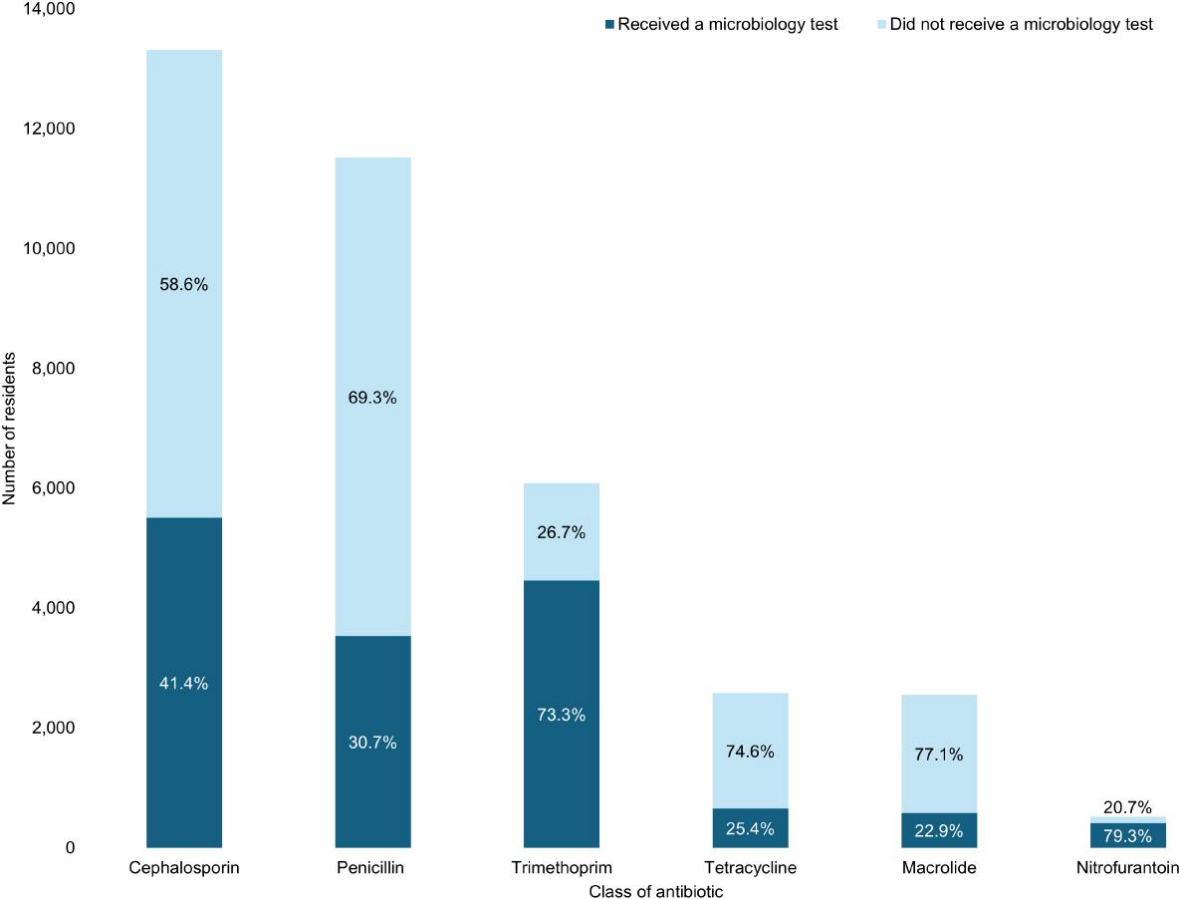
Number and proportion of residents receiving a microbiology test around the time of antibiotic initiation, by antibiotic type. All antibiotic types were counted for *n* = 3458 (9.4%) individuals who initiated >1 type of antibiotic (by ATC code) on the same date. Lincosamides, quinolone, nitroimidazole, aminoglycosides and glycopeptides not displayed due to low prevalence of use (each class ≤1.3% of antibiotics dispensed) (Table S3).

Overall, 15 407 (41.7%) residents received a microbiology test around the time of antibiotic initiation (Table 2). Urine tests were the most frequently claimed test (*n* = 11 162 residents, 30.2%) followed by microbial nucleic acid amplification (*n* = 2587, 7.0%) and skin tests (*n* = 1983, 5.4%). Testing varied by antibiotic type, ranging from 22.9% among macrolide users (585/2551) to 79.3% (413/521) of nitrofurantoin users (Figure 2). Microbiology tests were more commonly ordered when ‘access’ antibiotics were initiated (*n* = 7588 residents, 46.6%) than ‘curb’ antibiotics (*n* = 7978, 37.7%). Most tests were conducted before or on the day of antibiotic initiation (*n* = 12 370 80.3%) (Figure S2).

**Figure 3.**
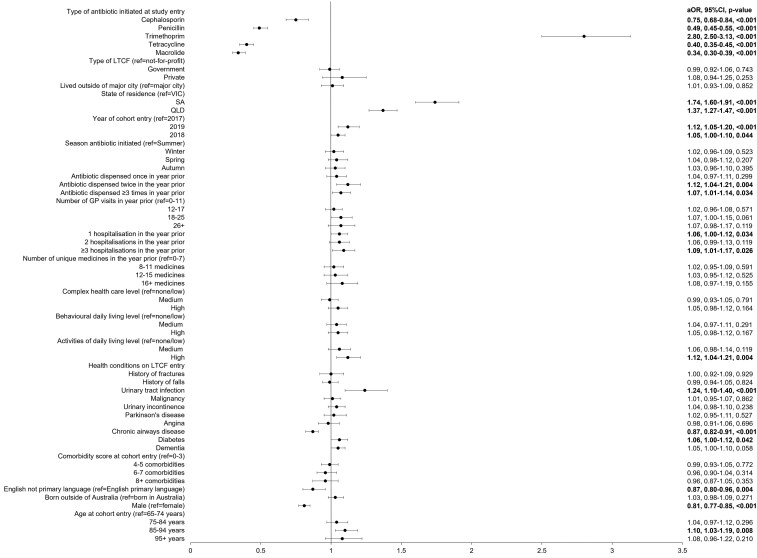
Adjusted odds ratios (aORs) with 95% confidence intervals (95%CIs) for logistic regression models evaluating factors associated with provision of a microbiology test around the time of antibiotic initiation. There were *n* = 611 (1.7%) residents with missing data who were excluded for complete case analysis. Nitrofurantoin, lincosamide, quinolones, nitroimidazole, aminoglycosides, glycopeptides and other systemic antibiotics were not included in the model due to low prevalence of use (each class <1.5% of antibiotics dispensed). Statistically significant results are shown in bold. GP, general medical practitioner, LTCF, long-term care facility, SA, South Australia, QLD, Queensland, VIC, Victoria.

Factors potentially associated with microbiology testing included a UTI at LTCF entry (aOR 1.24, 95%CI 1.10–1.40), more antibiotics dispensed in the year before initiation (aOR 1.12, 95%CI 1.04–1.21) or initiation of trimethoprim (aOR 2.80, 95%CI 2.50–3.13) (Figure 3). Residents living in LTCFs in South Australia (aOR 1.74, 95%CI 1.60–1.91) or Queensland (aOR 1.37, 95%CI 1.27–1.47) had higher odds of testing compared with those in Victoria. Males (aOR 0.81, 95%CI 0.77–0.85), those who did not speak English primarily (aOR 0.87, 95%CI 0.80–0.96), had airways disease (aOR 0.87, 95%CI 0.82–0.91) or prescribed a cephalosporin (aOR 0.75, 95%CI 0.68–0.84) or penicillin (aOR 0.49, 0.45–0.55) had lower odds of testing. Residents with urinary incontinence (aOR 1.08, 95%CI 1.01–1.15), diabetes (aOR 1.11, 95%CI 1.04–1.19) or dementia (aOR 1.10, 95%CI 1.04–1.17), or higher activities of daily living (aOR 1.21, 95%CI 1.11–1.32) or behavioural needs (aOR 1.13, 95%CI 1.05–1.22) had higher odds of urine testing (Figure S4).

**Figure 4.**
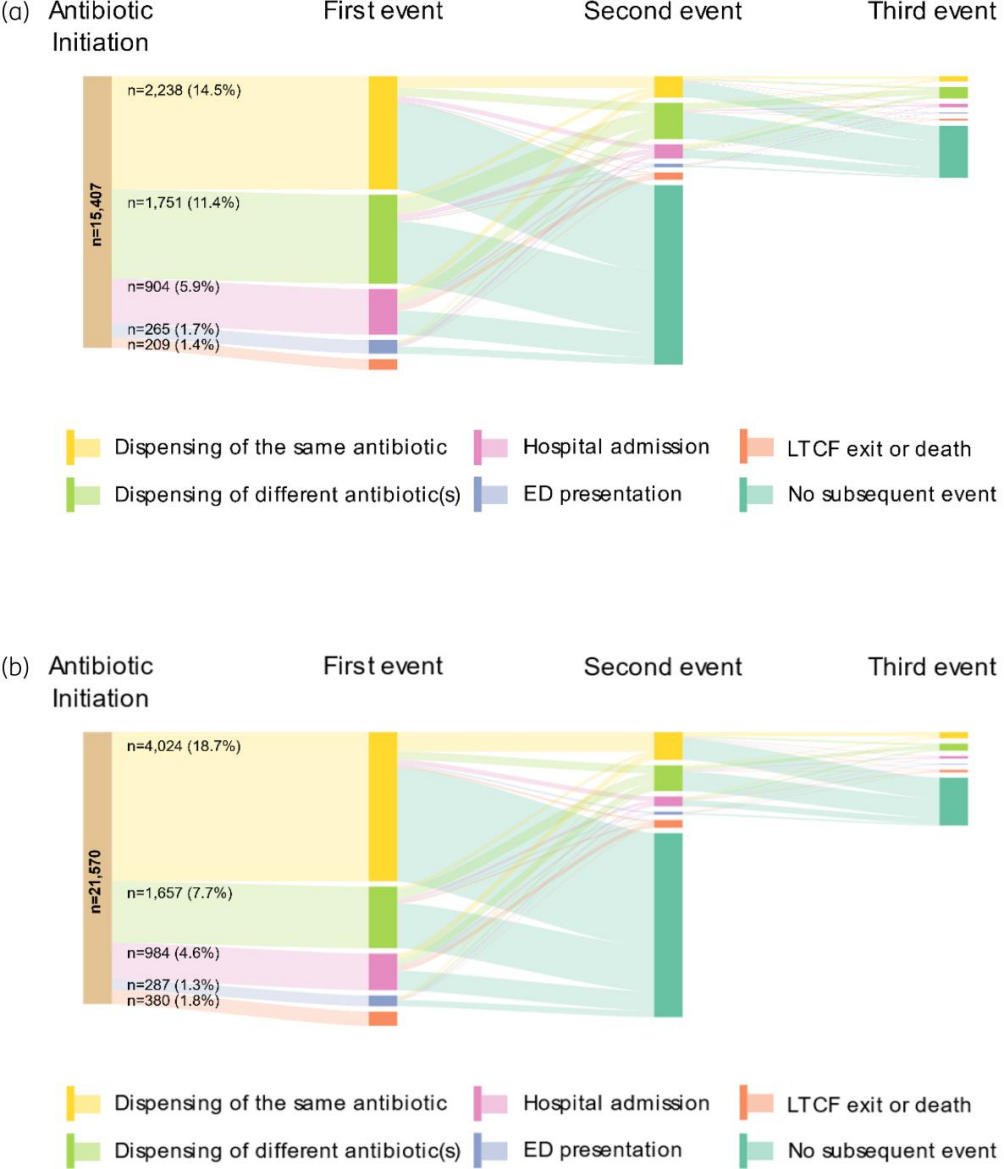
Treatment pathways in the 14 days after antibiotic initiation among individuals who (a) received a microbiology test around the time of antibiotic initiation (*n* = 15 407); and (b) did not receive a microbiology test (*n* = 21 570). Individuals who did not experience an event in the 14-day follow-up period are not shown. Events experienced after the third event and within 14 days were examined but are not displayed in the figure due to low prevalence (*n* = 256, 2.0% of people with ≥1 event). The width of pathways plotted for 1–5 individuals were determined as the width of three individuals for statistical disclosure control. If >1 antibiotic was dispensed on the date of study entry and another antibiotic was supplied in the 14-day follow-up period the resident was considered to have received the ‘same antibiotic’ if it was the same as at least one of those supplied at study entry, or a ‘different antibiotic’ if it was different to all of the antibiotics supplied initially. ED, emergency department, LTCF, long-term care facility.

Overall, 12 699 residents (34.3% of the overall cohort) had ongoing treatment over the following 14 days (Figure S3, Table S5) including 5367 residents who received a microbiology test (34.8% of the 15 407 residents tested) and 7332 residents who were not tested (34.0% of the 21 570 residents not tested). Among those tested, 14.5% were resupplied the same antibiotic, 11.4% were dispensed a different antibiotic and 8.9% were hospitalized, presented to the ED or died/exited care, as the first event in the treatment pathway (Figure 4). For residents not tested, 18.7% were resupplied the antibiotic as the first event in the treatment pathway, 7.7% received a different antibiotic and 7.7% were admitted to hospital, presented to ED or died/exited care. Overall, re-supply of the same or a different antibiotic were the most common events occurring in the 14 days after antibiotic initiation (Table S5). The most common reasons for ED presentation, hospitalization or death in the overall cohort are summarized in Table S6.

## Discussion

This large, population-based study of 36 977 individuals found only 4 in 10 residents initiating a systemic antibiotic after LTCF entry received a microbiology test around the time of initiation. Residents initiating trimethoprim or with a UTI on entry had higher odds of testing, which aligns with the high proportion of urine tests (72%) performed, while those with chronic airways disease were less likely to be tested. Approximately one-third of residents who started an antibiotic received further treatment (with the same antibiotic resupplied to 14.5% of residents tested and 18.7% of residents not tested) or had a hospital-related event or exited care within 2 weeks.

Although not all infections require testing, our finding that less than half of residents starting an antibiotic were tested suggests a high reliance on empiric or possible prophylactic therapy in LTCFs. Cephalosporins and penicillins are often recommended first-line for common infections in LTCFs and comprised ∼65% of antibiotics initiated. Our findings are broadly consistent with point-prevalence surveys elsewhere.^5,8,12,16,26–28^ In 2022, approximately one in five antibiotic prescriptions were for prophylactic use in Australian LTCFs,^12^ and in 2013 more than one-quarter were for prophylaxis in European LTCFs, with 21% of these having a related microbiological sample.^16^ Other studies have similarly concluded that empiric therapy is common in LTCFs,^29,30^ including a point-prevalence survey of 255 residents receiving an antibiotic in which only 5% were tested.^30^ A small cohort study (*n* = 181) of older people hospitalized in Spain with bacteraemic UTIs found inadequate empiric antibiotic treatment was doubled among residents of LTCFs compared with community-dwelling individuals (40% versus 21%).^31^ A previous study found that residents of LTCFs (*n* = 7388) were more likely to receive microbiology tests than those living in the community (*n* = 236 911), although only 25% of residents receiving an antibiotic with high potential for resistance were tested when it was initiated.^18^ Our finding that antibiotic selection changed for 11% of individuals with a microbiology test as the first step in their treatment pathway underscores the importance of appropriate testing to guide treatment decisions and minimize risk of hospitalizations and antibiotic resistance.^8,13,15,32^ In Australia, tests are required for some high risk first-line agents to receive subsidised treatments, such as quinolones. However, our study found only 59% of residents dispensed a quinolone were tested, suggesting improvements may be needed. As well as ensuring best possible clinical outcomes, optimizing microbiology testing may also inform stewardship initiatives, such as the development of antibiograms for local resistance data.^33^

The most common test provided in this study was a urine test, with ∼7 in 10 trimethoprim users and 8 in 10 nitrofurantoin users tested. Regression analyses found residents initiating trimethoprim or with a UTI on entry were more likely to be tested. The frequent use of microbiology tests among trimethoprim and nitrofurantoin users and higher odds of urine testing in our study suggests awareness of appropriate UTI management practices, which include avoiding routine screening (e.g. dipsticks) and treatment of asymptomatic bacteriuria^15,34^ to minimize antibiotic over use and harms.^35,36^ Our findings align with a previous Australian study that found most isolates collected in LTCFs were urinary.^33^ Other studies have reported that residents are more likely to have urine tests compared with community-dwelling indivdiuals,^18^ and 26.5% of residents admitted to hospital for infection had a urinalysis or dipstick prior to hospital transfer.^32^ These findings are not unexpected given UTIs are common reasons for antibiotic use in LTCFs,^12,28,34,36^ with 50%–66% of antimicrobials used on a single day prescribed for the treatment or prevention of UTIs (commonly trimethoprim, cefalexin and nitrofurantoin).^28^ In 2025, Australia’s Antibiotic Therapeutic Guideline was updated and recommends nitrofurantoin as first-line for acute cystitis, with trimethoprim third-line (29% of *Escherichia coli* among residents of Australian LTCFs was resistant to trimethoprim^37^).^38^ Thus, in light of these recent changes, ongoing monitoring of UTI management, including microbiology testing, is suggested. Improving both diagnostic and antimicrobial stewardship remains an area of focus for this setting, which must be balanced with the potential for over use of tests. Existing evidence suggests multifaceted interventions that incorporate audit and feedback, education and reminders,^39^ and quality indicators for UTI diagnosis and treatment^40,41^ could be considered.

Our population-level study is among few other studies to examine microbiology testing and treatment pathways. We found one-third of tested residents received further treatment, had a hospital-related event or exited care within 2 weeks. Several contextual factors may have influenced the low rate of testing and subsequent treatment pathways observed. Infectious diseases physicians do not consult on-site in Australian LTCFs and 96% of antibiotics were prescribed by general medical practitioners, who typically visit LTCFs periodically, include locums, and may prescribe remotely.^27,36^ There are also known delays in collecting samples, and receiving and reviewing microbiology test results in LTCFs, especially in non-metropolitan areas.^32^ Respiratory infections were the most common reason for unplanned hospitalization during our follow-up, however, our study found low use of respiratory tests (1%) and lower odds of testing among residents with airways disease. Respiratory tract infections often cannot be confirmed by microbiology testing (e.g. non-productive cough, sputum unavailable) and some microbial nucleic acid amplification tests may be used to identify respiratory tract infections. Where appropriate, timely microbiology testing is important to optimize infection resolution (i.e. reduces length of stay for people hospitalized with pneumonia^42^), although these findings may reflect challenges with obtaining adequate respiratory secretions and uncontaminated samples from older people.^14,43^ Australian LTCF accreditation standards require facilities to implement IPC mechanisms such as antimicrobial stewardship initiatives and have a dedicated IPC lead role (and/or IPC team) on-site.^13,15,44,45^ However, the extent to which this is operationalized in the LTCF probably varies and pharmacists, who can support these initiatives, have only recently begun working on-site in Australia.^46^

### Strengths and limitations

This population-level study comprehensively examined microbiology testing and treatment pathways following antibiotic initiation in LTCFs. We included individuals entering LTCFs in three Australian states (comprising 54% of the national population^6^) and thus findings are likely generalizable to the national population of older people accessing LTCFs. Our 21-day screening window for microbiology testing allowed sufficient time for samples to be taken, processed and results to be acted on. Exclusion of individuals with a recent hospitalization reduced bias in ascertaining tests, as those provided in hospitals are not MBS-subsidised, and antibiotic use in hospital is only PBS-subsidised on discharge. We included individuals entering LTCFs permanently during a 30-month period (from 2017 to mid-2019) who initiated an antibiotic. New residents of LTCFs can have different experiences and care needs compared with individuals who have lived in a LTCF for longer and it is also possible that treatment recommendations, guidelines and local practices may have changed over this time. Our study examined microbiology tests and treatment pathways following any systemic antibiotic initiation in LTCFs, thus individuals who were tested and did not initiate a systemic antibiotic are not included. Current pathology testing subsidies in Australia only permit a maximum of three pathology tests claimed per person per day. The three most expensive pathology tests are usually claimed. Antibiotics and tests supplied privately, and any further tests provided above the subsidised threshold, were not visible in the datasets: thus, some test types may be under-estimated. Test results and their influence on treatment pathways, infection resolution, duration of antibiotic use and appropriateness of microbiology testing and antibiotic prescribing were also unable to be ascertained from the datasets. Differences between the date of antibiotic initiation or prescribing (e.g. if the antibiotic was supplied from a LTCF imprest, or dispensed for packing into a dose administration aid^27^) and the date of dispensing were examined and were negligible (median 0 days, IQR 0–0). We could not examine testing around the time of topical antibiotic use (which comprises 40% of all antimicrobials prescribed in Australian LTCFs^12^) because few topical antibiotics are PBS-subsidised.

### Conclusion

Microbiology testing is an integral component of antimicrobial stewardship. However, our population-level study found ∼60% of residents of LTCFs commence an antibiotic without a microbiology test being performed. This highlights an opportunity to improve the provision of microbiology tests among residents of LTCFs. Our findings show a reliance on empirical antibiotic therapy in LTCFs, and microbiology tests are important for guiding antibiotic selection and effective treatment of infections. Our findings further support the need for LTCF-specific initiatives to optimize microbiology testing. Expanding current policy and practice requirements, including antimicrobial stewardship initiatives and availability of local resistance data, is important to ensure judicious use of tests and antibiotics in LTCFs.

## Supplementary Material

dkag212_Supplementary_Data

## Supplementary data

Figures S1 to S4 and Tables S1 to S6 are available as Supplementary data at *JAC* Online.
