# Supplementary material for: Microbiology testing around the time of antibiotic initiation among residents of long-term care facilities
Source: J Antimicrob Chemother. 2026 Jun 29;81(7):dkag212. doi: 10.1093/jac/dkag212 (PMC13311677; doi:10.1093/jac/dkag212)
Supplement: dkag212_Supplementary_Data [file dkag212_supplementary_data.docx]

# SUPPLEMENTARY MATERIALS

**Table S1.** World Health Organisation Anatomical Therapeutic Chemical (ATC) codes for each antibiotic, by antibiotic class and Australia’s Priority Antibacterial List for Antimicrobial Resistance Containment classification.

**Table S2.** List of Medicare Benefits Schedule (MBS) item numbers to ascertain provision of microbiology tests.

**Table S3.** Number and proportion of antibiotics dispensed on the date of initiation, by antibiotic type.

**Table S4.** Number of individuals who initiated an antibiotic and were provided microbiology tests around the time of antibiotic initiation, by Australia’s Priority Antibacterial List for Antimicrobial Resistance Containment classification.

**Table S5.** Treatment pathways in the 14 days after antibiotic initiation among (i) all individuals (n=36,977), (ii) individuals who received a microbiology test around the time of antibiotic initiation (n=15,407), and (iii) those who did not receive a microbiology test (n=21,570).

**Table S6.** Primary reason for (i) presentation to an emergency department, (ii) hospitalisation, or (iii) death for individuals who experienced these events in the 14 days after antibiotic initiation.

**Figure S1.** Diagram summarising the study design.

**Figure S2.** Scatter plot of the proportion of residents who received a microbiology test around the time of antibiotic initiation that was during the 14 days before and after antibiotic initiation.

**Figure S3.** Treatment pathways in the 14 days after antibiotic initiation (n=12,699).

**Figure S4.** Adjusted odds ratio with 95% confidence intervals for logistic regression models evaluating resident characteristics associated with provision of a urine test around the time of antibiotic initiation.

**Table S1.** World Health Organisation (WHO) Anatomical Therapeutic Chemical (ATC) codes for each antibiotic, by antibiotic class and Australia’s Priority Antibacterial List for Antimicrobial Resistance Containment classification.

| **Class** | **Antibiotic** | **Priority class** | **WHO ATC code** |
| --- | --- | --- | --- |
| Cephalosporin | Cefalexin | Curb | J01DB01 |
|  | Cefalotin | Curb | J01DB03 |
|  | Cefazolin | Curb | J01DB04 |
|  | Cefuroxime | Curb | J01DC02 |
|  | Cefaclor | Curb | J01DC04 |
|  | Cefotaxime | Curb | J01DD01 |
|  | Ceftriaxone | Curb | J01DD04 |
|  | Cefepime | Contain | J01DE01 |
| Penicillin | Ampicillin | Access | J01CA01 |
|  | Amoxicillin | Access | J01CA04 |
|  | Benzylpenicillin | Access | J01CE01 |
|  | Phenoxymethylpenicillin | Access | J01CE02 |
|  | Benzathine benzylpenicillin | Access | J01CE08 |
|  | Procaine benzylpenicillin | Access | J01CE09 |
|  | Benzathine phenoxymethylpenicillin | Unassigned* | J01CE10 |
|  | Dicloxacillin | Access | J01CF01 |
|  | Flucloxacillin | Access | J01CF05 |
|  | Amoxicillin and beta-lactamase inhibitor | Curb | J01CR02 |
|  | Ticarcillin and beta-lactamase inhibitor | Curb | J01CR03 |
| Trimethoprim | Trimethoprim | Access | J01EA01 |
|  | Trimethoprim with sulfamethoxazole | Access | J01EE01 |
| Tetracycline | Doxycycline | Access | J01AA02 |
|  | Tetracycline | Access | J01AA07 |
|  | Minocycline | Access | J01AA08 |
| Macrolide | Erythromycin | Curb | J01FA01 |
|  | Roxithromycin | Curb | J01FA06 |
|  | Clarithromycin | Curb | J01FA09 |
|  | Azithromycin | Curb | J01FA10 |
| Nitrofurantoin | Nitrofurantoin | Access | J01XE01 |
| Lincosamide | Clindamycin | Curb | J01FF01 |
|  | Lincomycin | Curb | J01FF02 |
| Quinolone | Ciprofloxacin | Curb | J01MA02 |
|  | Norfloxacin | Curb | J01MA06 |
| Nitroimidazole | Metronidazole | Access | J01XD01 |
|  | Tinidazole | Access | J01XD02 |
| Aminoglycoside | Tobramycin | Access | J01GB01 |
|  | Gentamicin | Access | J01GB03 |
| Glycopeptide | Vancomycin | Curb | J01XA01 |
|  | Vancomycin and colistin | Curb | A07AA09 |
| Other systemic antibiotics | Fusidic acid | Curb | J01XC01 |
|  | Isoniazid | Unassigned* | J04AC01 |
|  | Rifaximin | Curb | A07AA11 |
|  | Dapsone | Curb | J04BA02 |

*Antibiotics that are not assigned an Antimicrobial Resistance Containment classification (i.e., benzathine phenoxymethylpenicillin, isoniazid) and assigned contain (cefepime) were included as curb antibiotics in analyses.

**Table S2.** List of Medicare Benefits Schedule (MBS) item numbers to ascertain provision of microbiology tests.

| **Category** | **MBS Item numbers** | **MBS Item Descriptions** |
| --- | --- | --- |
| **Urine** | 69333 | Urine examination (including serial examinations) by any means other than simple culture by dip slide. |
|  | 73805, 73832 | Microscopy of urine, excluding dipstick testing.  Microscopy of urine, excluding dipstick testing by a participating nurse practitioner. |
| **Skin/superficial** | 69306 | Microscopy and culture to detect pathogenic micro-organisms from skin or other superficial sites; 1 or more tests on 1 or more specimens. |
|  | 69309 | Microscopy and culture to detect dermatophytes and other fungi causing cutaneous disease from skin scrapings, skin biopsies, hair and nails (excluding swab specimens); 1 or more tests on 1 or more specimens. |
|  | 73810, 73837 | Microscopy for fungi in skin, hair or nails -1 or more sites / Microscopy for fungi in skin, hair or nails by a participating nurse practitioner; 1 or more sites |
| **Faecal** | 69345 | Culture and (if performed) microscopy without concentration techniques of faeces for faecal pathogens, using at least 2 selective or enrichment media and culture in at least 2 different atmospheres; 1 examination in any 7-day period. |
| **Respiratory** | 69318 | Microscopy and culture to detect pathogenic micro-organisms from specimens of sputum; 1 or more tests on 1 or more specimens. |
| **Genital** | 69312 | Microscopy and culture to detect pathogenic micro-organisms from urethra, vagina, cervix, or rectum (except for faecal pathogens); 1 or more tests on 1 or more specimens. |
| **Eye/Ear/Nose/Throat (EENT)** | 69303 | Culture and (if performed) microscopy to detect pathogenic micro-organisms from nasal swabs, throat swabs, eye swabs and ear swabs (excluding swabs taken for epidemiological surveillance), including (if performed):  (a) pathogen identification and antibiotic susceptibility testing; or  (b) a service described in item 69300; specimens from 1 or more sites |
| **Blood** | 69354, 69357, 69360 | Blood culture for pathogenic micro-organisms (other than viruses), including sub-cultures and (if performed): (a) identification of any cultured pathogen; and (b) necessary antibiotic susceptibility testing; to a maximum of 3 sets of cultures |
| **Targeted** |  |  |
| *Mycobacteria* | 69324, 69325, 69327, 69328, 69330, 69331 | Microscopy (with appropriate stains) and culture for mycobacteria - 1 specimen of sputum, urine, or other body fluid or 1 operative or biopsy specimen; 1 or more tests on 1 or more specimens |
| *Chlamydia trachomatis* | 69315, 69369, 69370  69316, 69317, 69319 | Detection of Chlamydia trachomatis by any method - 1 test (Item is subject to rule 26); 1 or more tests on 1 or more specimens.(69319=2 tests described in item 69494 and a test described in 69316. (Item is subject to rule 26) |
| *Cryptosporidium/Giardia* | 69336**,** 69339 | Microscopy of faeces for ova, cysts and parasites that must include a concentration technique, and the use of fixed stains or antigen detection for cryptosporidia and giardia. |
| *Clostridioides difficile* | 69363 | Detection of Clostridioides difficile or Clostridioides difficile toxin (except if a service described in item 69345 has been performed); one or more tests. |
| *Hepatitis virus* | 69444,69445, 69451, 69475, 69478, 69481, 69482, 69483, 69484, 69488, 69489, 69491, 69492, 69499, 69500 | Detection of Hepatitis C viral RNA in a patient undertaking antiviral therapy for chronic HCV hepatitis (including a service described in item 69499); 1 or more tests |
| *Epstein Barr Virus* | 69472, 69474 | Detection of antibodies to Epstein Barr Virus using specific serology; 1 or more tests |
| **Microbial nucleic acid amplification*** | 69300 | Microscopy of wet film material other than blood, from 1 or more sites, obtained directly from a patient (not cultures) including:  (a) differential cell count (if performed); or  (b) examination for dermatophytes; or  (c) dark ground illumination; or  (d) stained preparation or preparations using any relevant stain or stains;1 or more tests |
|  | 69321 | Microscopy and culture of post-operative wounds, aspirates of body cavities, synovial fluid, CSF or operative or biopsy specimens, for the presence of pathogenic micro-organisms involving aerobic and anaerobic cultures and the use of different culture media, and including (if performed):  (a) pathogen identification and antibiotic susceptibility testing; or  (b) a service described in item 69300, 69303, 69306, 69312 or 69318; specimens from 1 or more sites. |
|  | 69399, 69402  69384, 69387, 69390, 69393, 69396, 69400, 69401 | Quantitation of 1 antibody to microbial antigens not elsewhere described in the Schedule, one or more tests. |
|  | 69364, 69365, 69367, 69372, 69373, 69374, 69375, 69376  69494, 69495, 69496, 69497, 69498 | Detection of a virus or microbial antigen or microbial nucleic acid (not elsewhere specified);1 or more tests (Item is subject to rule 6 and 26) |
|  | 73807, 73834 | Microscopy for wet film other than urine, including any relevant stain.  Microscopy for wet film other than urine, including any relevant stain by a participating nurse practitioner. |
|  | 73808, 73835 | Microscopy of Gram-stained film, including (if performed) a service described in item 73805 or 73807 Microscopy of Gram-stained film, including (if performed) a service described in item 73832 or 73834 by a participating nurse practitioner. |

*Microbial nucleic acid amplification tests also include some tests that are not specific to other test types.

**Table S3.** Number and proportion of antibiotics dispensed on the date of initiation, by antibiotic type.

| **Type of antibiotic** | **Number and proportion of antibiotics dispensed at study entry** |
| --- | --- |
| Any antibiotic | 40 734 (100.0) |
| Cephalosporins | 14 355 (35.2) |
| Penicillins | 12 525 (30.7) |
| Trimethoprim | 6 311 (15.5) |
| Tetracyclines | 2 852 (7.0) |
| Macrolides | 2 835 (7.0) |
| Nitrofurantoin | 531 (1.3) |
| Lincosamides | 491 (1.2) |
| Quinolones | 400 (1.0) |
| Nitroimidazoles | 358 (0.9) |
| Aminoglycosides | 37 (0.1) |
| Glycopeptides | 6 (0.0) |
| Other systemic antibiotics | 33 (0.1) |

Different types of antibiotics determined by unique WHO ATC codes. ‘Other systemic antibiotics’ includes fusidic acid, isoniazid, rifaximin and dapsone.

**Table S4.** Number of individuals who initiated an antibiotic and were provided microbiology tests around the time of antibiotic initiation, by Australia’s Priority Antibacterial List for Antimicrobial Resistance Containment classification.

| **Residents receiving microbiology tests around the time of antibiotic initiation**  **n (%)** | **Any** | **Access antibiotic** | **Curb antibiotic*** |
| --- | --- | --- | --- |
| Antibiotic initiation | 36977 (100.0) | 16280 (44.0) | 21145 (57.2) |
| Any microbiology test | 15407 (41.7) | 7588 (46.6) | 7978 (37.7) |
| ***Type of microbiology test*** |  |  |  |
| Urine | 11162 (30.2) | 5888 (36.2) | 5357 (25.3) |
| Skin/superficial | 1983 (5.4) | 628 (3.9) | 1376 (6.5) |
| Respiratory | 393 (1.1) | 192 (1.2) | 216 (1.0) |
| Faecal | 322 (0.9) | 180 (1.1) | 151 (0.7) |
| Genital | 140 (0.4) | 79 (0.5) | 65 (0.3) |
| Eye/Ear/Nose/Throat | 193 (0.5) | 84 (0.5) | 111 (0.5) |
| Blood | 178 (0.5) | 72 (0.4) | 116 (0.5) |
| Targeted |  |  |  |
| *Mycobacteria* | 23 (0.1) | 8 (0.0) | 17 (0.1) |
| *Chlamydia trachomatis* | 13 (0.0) | 7 (0.0) | 6 (0.0) |
| *Cryptosporidum/Giardia* | 183 (0.5) | 100 (0.6) | 87 (0.4) |
| *Clostridioides difficile* | 19 (0.1) | 6 (0.0) | 13 (0.1) |
| *Hepatitis virus* | 67 (0.2) | 30 (0.2) | 37 (0.2) |
| *Epstein Barr virus* | n/a | n/a | n/a |
| Microbial nucleic acid amplification† | 2587 (7.0) | 1174 (7.2) | 1458 (6.9) |

All antibiotics were counted for n=3,458 (9.4%) individuals who initiated >1 antibiotic (by ATC code) on the same date.

*The classification of Curb antibiotics includes cefepime (Contain antibiotic, n=1-5) and two antibiotics (benzathine phenoxymethylpenicillin and isoniazid, n=1-5) that were not assigned a priority class (**Table S1**).

†Microbial nucleic acid amplification tests also include some tests that are not specific to other test types.

**Table S5.** Treatment pathways in the 14 days after antibiotic initiation among (i) all individuals (n=36,977), (ii) individuals who received a microbiology test around the time of antibiotic initiation (n=15,407), and (iii) those who did not receive a microbiology test (n=21,570).

| **Event type** | ***To***  **Dispensed the same antibiotic**  **[n,%]** | ***To***  **Dispensed a different antibiotic**  **[n,%]** | ***To***  **Emergency department presentation [n,%]** | ***To***  **Hospital admission**  **[n,%]** | ***To***  **LTCF exit or death**  **[n,%]** |
| --- | --- | --- | --- | --- | --- |
| **All individuals in study cohort (n=36,977)** | | | | | |
| Overall (n=36,977)* | 6,760 (18.3) | 4,386 (11.9) | 697 (1.9) | 2,454 (6.6) | 1,067 (2.9) |
| First event (n=12,699) | 6,262 (16.9) | 3,408 (9.2) | 552 (1.5) | 1,888 (5.1) | 589 (1.6) |
| Second event  Total (n=3,607) | 1,168 (3.2) | 1,405 (3.8) | 143 (0.4) | 540 (1.5) | 351 (0.9) |
| *From* Dispensed the same antibiotic | 759 (2.1) | 392 (1.1) | 58 (0.2) | 219 (0.6) | 72 (0.2) |
| *From* Dispensed a different antibiotic | 133 (0.4) | 567 (1.5) | 39 (0.1) | 171 (0.5) | 65 (0.2) |
| *From* Emergency department presentation | 54 (0.1) | 68 (0.2) | 19 (0.1) | 74 (0.2) | 28 (0.1) |
| *From* Hospital admission | 222 (0.6) | 378 (1.0) | 27 (0.1) | 76 (0.2) | 186 (0.5) |
| Third event  Total (n=943) | 270 (0.7) | 415 (1.1) | 33 (0.1) | 132 (0.4) | 93 (0.3) |
| *From* Dispensed the same antibiotic | 161 (0.4) | 82 (0.2) | 8 (0.0) | 32 (0.1) | 23 (0.1) |
| *From* Dispensed a different antibiotic | 47 (0.1) | 216 (0.6) | 13 (0.0) | 62 (0.2) | 25 (0.1) |
| *From* Emergency department presentation | 7 (0.0) | 16 (0.0) | 7-11† | 25 (0.1) | 14 (0.0) |
| *From* Hospital admission | 55 (0.1) | 101 (0.3) | 1-5† | 13 (0.0) | 31 (0.1) |
| **Individuals who received a microbiology test around the time of antibiotic initiation (n=15,407)** | | | | | |
| Overall (n=15,407)* | 2,477 (16.1) | 2,222 (14.4) | 328 (2.1) | 1,200 (7.8) | 399 (2.6) |
| First event (n=5,367) | 2,238 (14.5) | 1,751 (11.4) | 265 (1.7) | 904 (5.9) | 209 (1.4) |
| Second event  Total (n=1,618) | 419 (2.7) | 712 (4.6) | 64 (0.4) | 278 (1.8) | 145 (0.9) |
| *From* Dispensed the same antibiotic | 233 (1.5) | 174 (1.1) | 21 (0.1) | 93 (0.6) | 20 (0.1) |
| *From* Dispensed a different antibiotic | 71 (0.5) | 309 (2.0) | 17 (0.1) | 101 (0.7) | 26 (0.2) |
| *From* Emergency department presentation | 22 (0.1) | 31 (0.2) | 10 (0.1) | 48 (0.3) | 15 (0.1) |
| *From* Hospital admission | 93 (0.6) | 198 (1.3) | 16 (0.1) | 36 (0.2) | 84 (0.5) |
| Third event  Total (n=447) | 105 (0.7) | 227 (1.5) | 17 (0.1) | 67 (0.4) | 31 (0.2) |
| *From* Dispensed the same antibiotic | 51 (0.3) | 39 (0.3) | 1-5† | 15 (0.1) | 8 (0.1) |
| *From* Dispensed a different antibiotic | 21 (0.1) | 120 (0.8) | 7 (0.0) | 32 (0.2) | 12 (0.1) |
| *From* Emergency department presentation | 1-5† | 6 (0.0) | 1-5† | 15-19† | 1-5† |
| *From* Hospital admission | 28-32† | 62 (0.4) | 1-5† | 1-5† | 6-10† |
| **Individuals who did not receive a microbiology test around the time of antibiotic initiation (n=21,570)** | | | | | |
| Overall (n=21,570)* | 4283 (19.9) | 2164 (10.0) | 369 (1.7) | 1,254 (5.8) | 668 (3.1) |
| First event (n=7,332) | 4024 (18.7) | 1657 (7.7) | 287 (1.3) | 984 (4.6) | 380 (1.8) |
| Second event  Total (n=1,989) | 749 (3.5) | 693 (3.2) | 79 (0.4) | 262 (1.2) | 206 (1.0) |
| *From* Dispensed the same antibiotic | 526 (2.4) | 218 (1.0) | 37 (0.2) | 126 (0.6) | 52 (0.2) |
| *From* Dispensed a different antibiotic | 62 (0.3) | 258 (1.2) | 22 (0.1) | 70 (0.3) | 39 (0.2) |
| *From* Emergency department presentation | 32 (0.1) | 37 (0.2) | 9 (0.0) | 26 (0.1) | 13 (0.1) |
| *From* Hospital admission | 129 (0.6) | 180 (0.8) | 11 (0.1) | 40 (0.2) | 102 (0.5) |
| Third event  Total (n=496) | 165 (0.8) | 188 (0.9) | 16 (0.1) | 65 (0.3) | 62 (0.3) |
| *From* Dispensed the same antibiotic | 110 (0.5) | 43 (0.2) | 1-5† | 17 (0.1) | 15 (0.1) |
| *From* Dispensed a different antibiotic | 26 (0.1) | 96 (0.4) | 6 (0.0) | 30 (0.1) | 13 (0.1) |
| *From* Emergency department presentation | 1-5† | 10 (0.0) | 1-5† | 10 (0.0) | 13 (0.1) |
| *From* Hospital admission | 24-28† | 39 (0.2) | 1-5† | 8 (0.0) | 21 (0.1) |

*The number and proportion of people who experienced at least one event of interest during the 14 days after antibiotic initiation.

†Number presented as a range due to low counts (≤5 residents) and disclosure control.

If >1 antibiotic was dispensed on the date of study entry and another antibiotic was supplied in the 14-day follow-up period the resident was considered to have received the ‘same antibiotic’ if it was the same as at least one of those supplied at study entry, or a ‘different antibiotic’ if it was different to all of the antibiotics supplied initially.

**Table S6.** Primary reason for (i) presentation to an emergency department, (ii) hospitalisation, or (iii) death for individuals who experienced these events in the 14 days after antibiotic initiation.

| **Primary reason*** | **Emergency Department presentation**  **(n=694)**  **[n,%]** | **Hospital admission (n=2,452)**  **[n,%]** | **Death (n=976)**  **[n,%]** |
| --- | --- | --- | --- |
| Diseases of the respiratory system e.g., respiratory infection, chronic airways disease [J00-J99] | 72 (10.4) | 523 (21.3) | 154 (15.8) |
| Symptoms, signs and abnormal clinical findings, not elsewhere classified e.g., tendency to fall, malaise and fatigue, syncope and collapse [R00-R99] | 182 (26.2) | 470 (19.2) | 8 (0.8) |
| Injury, poisoning and certain other consequences of external causes e.g., fractures, dislocation, sprain and strain, open wound [S00-T98] | 170 (24.5) | 340 (13.9) | 0 (0.0) |
| Diseases of the circulatory system e.g., heart failure, stroke, myocardial infarction [I00-I99] | 40 (5.8) | 222 (9.1) | 241 (24.7) |
| Neoplasms [C00-D48] | 1-5† | 17-21† | 240 (24.6) |
| Diseases of the genitourinary system e.g., urinary tract infection, acute kidney failure, chronic kidney disease [N00-N99] | 36 (5.2) | 169 (6.9) | 36 (3.7) |
| Mental and behavioural disorders e.g., delirium and/or dementia, depression, anxiety [F00-F99] | 18 (2.6) | 117 (4.8) | 95 (9.7) |
| Diseases of the digestive system e.g., gastritis, gastric ulcer, inflammatory bowel disease, liver disease [K00-K99] | 18 (2.6) | 116 (4.7) | 25 (2.6) |
| Diseases of the skin and subcutaneous tissue e.g., cellulitis, ulcer [L00-L99] | 36 (5.2) | 114 (4.6) | 8 (0.8) |
| Endocrine, nutritional and metabolic diseases e.g., type 2 diabetes mellitus and complications, malnutrition [E00-E99] | 16 (2.3) | 86 (3.5) | 35 (3.6) |
| Diseases of the nervous system e.g., Alzheimer’s disease, Parkinson’s disease [G00-G99] | 9 (1.3) | 36 (1.5) | 90 (9.2) |
| Certain infectious and parasitic diseases e.g., sepsis [A00-B99] | 18 (2.6) | 97 (4.0) | 9 (0.9) |
| Diseases of the musculoskeletal system and connective tissue e.g., arthritis, gout, osteoporosis [M00-M99] | 20 (2.9) | 43 (1.8) | 9 (0.9) |
| Diseases of the blood and blood-forming organs and certain disorders involving the immune mechanism e.g., anaemia [D50-D99] | 5-9† | 54 (2.2) | 1-5† |
| Factors influencing health status and contact with health services e.g., rehabilitation, awaiting placement to residential aged care service, need for assistant at home and no other household member able to render care, respite care [Z00-Z99] | 32 (4.6) | 23 (0.9) | 0 (0.0) |
| External causes of morbidity and mortality [V00-Y99] | 8 (1.2) | 13 (0.5) | 24 (2.5) |
| Diseases of the eye and adnexa e.g., glaucoma [H00-H59] | 1-5† | 5-9† | 0 (0.0) |
| Diseases of the ear and mastoid process e.g., otitis media, vertigo [H60-H99] | 1-5† | 1-5† | 1-5† |
| Congenital malformations, deformations and chromosomal abnormalities [Q00-Q99] | 0 (0.0) | 0 (0.0) | 0 (0.0) |

There are 19 events excluded from this table due to missing diagnoses.

*Primary reason determined by the International Classification of Diseases Tenth Revision, Australian Modification (ICD-10-AM) codes, categorised by chapter group.

†Number presented as a range due to low counts (≤5 residents) and disclosure control.

**Figure S1.** Diagram summarising the study design.

**
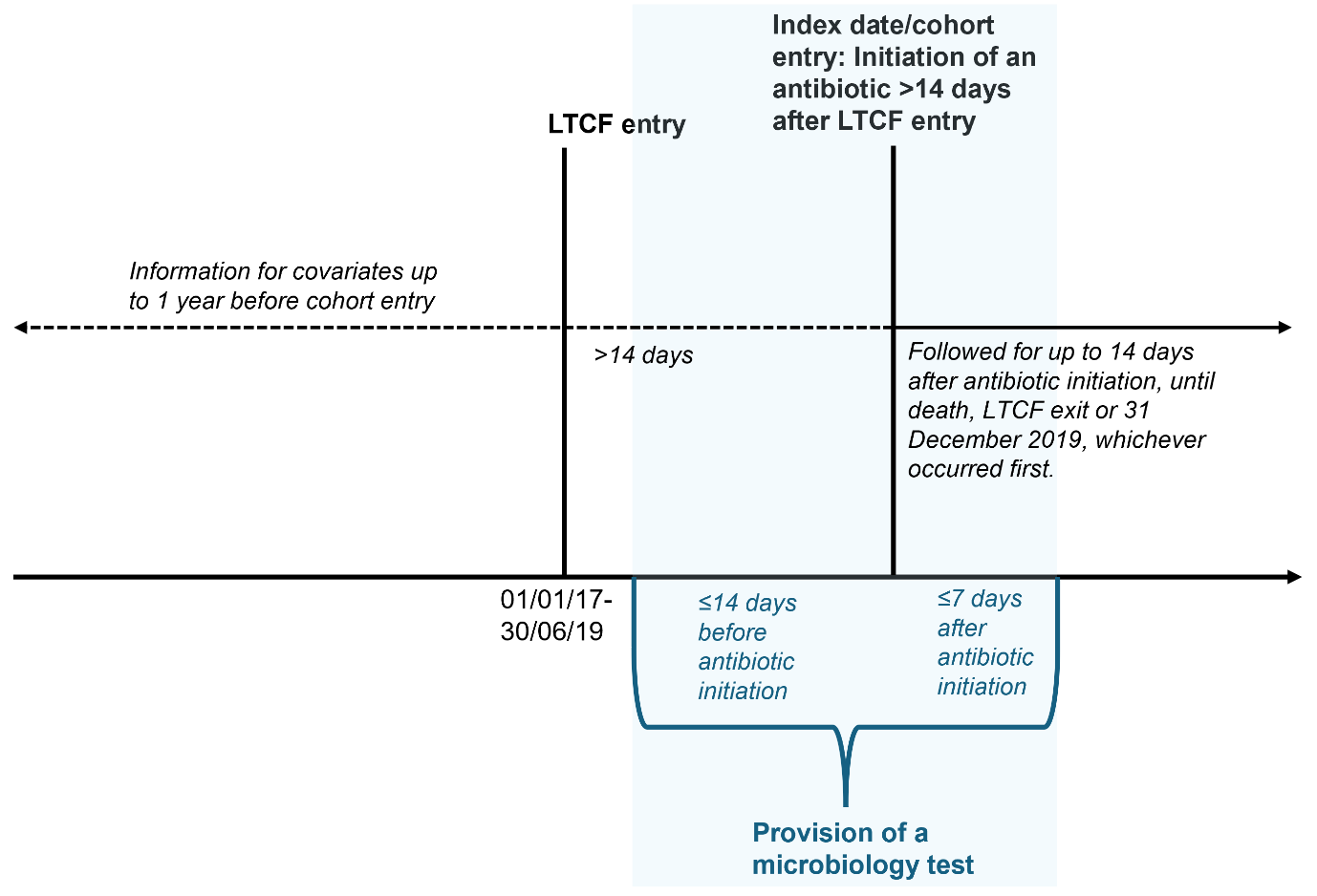
**

LTCF, long-term care facility.

**Figure S2.** Scatter plot of the proportion of residents who received a microbiology test around the time of antibiotic initiation that was during the 14 days before and after antibiotic initiation.


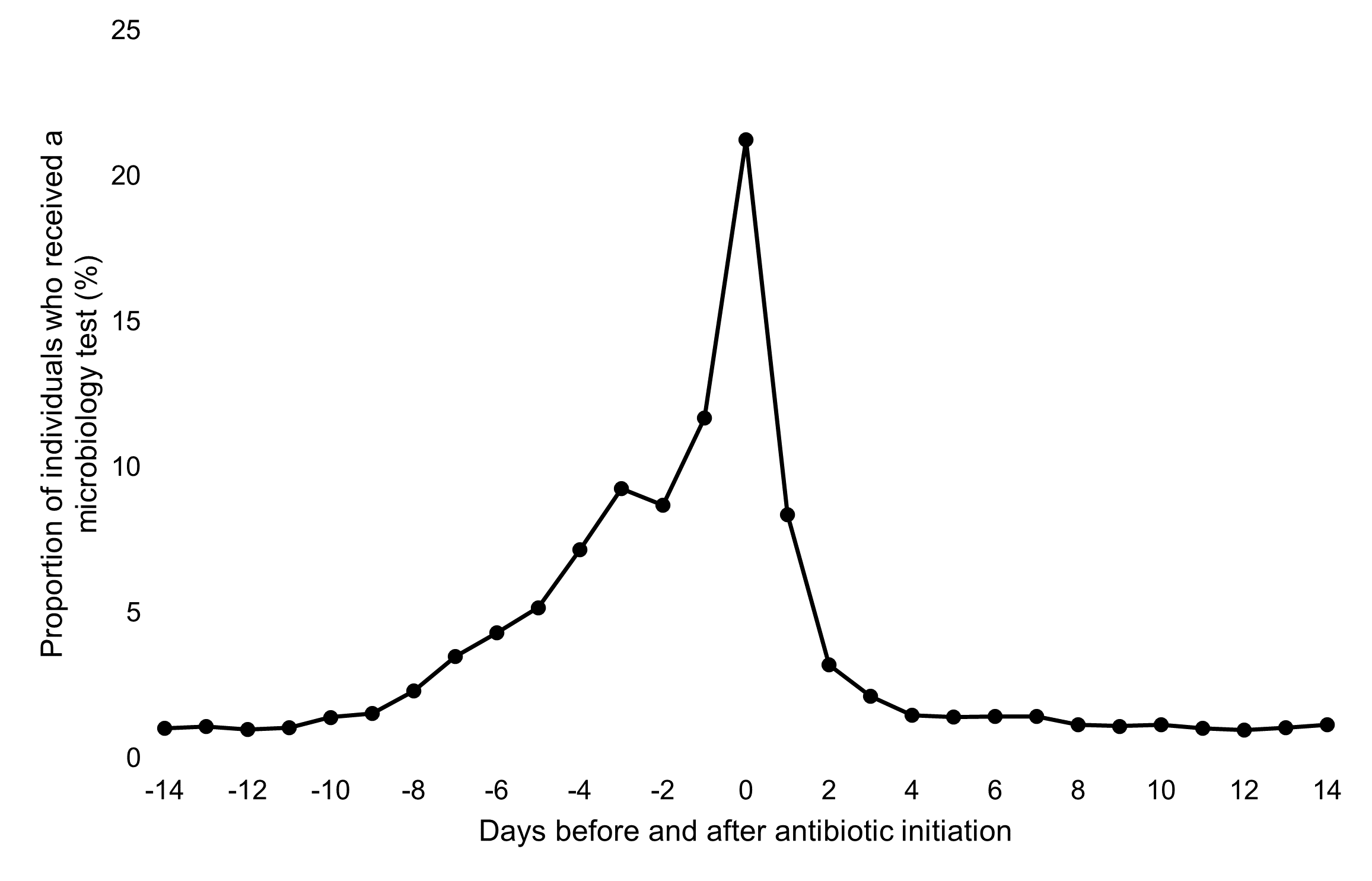


Day 0 is the date of antibiotic initiation.

**Figure S3.** Treatment pathways in the 14 days after antibiotic initiation (n=12,699).


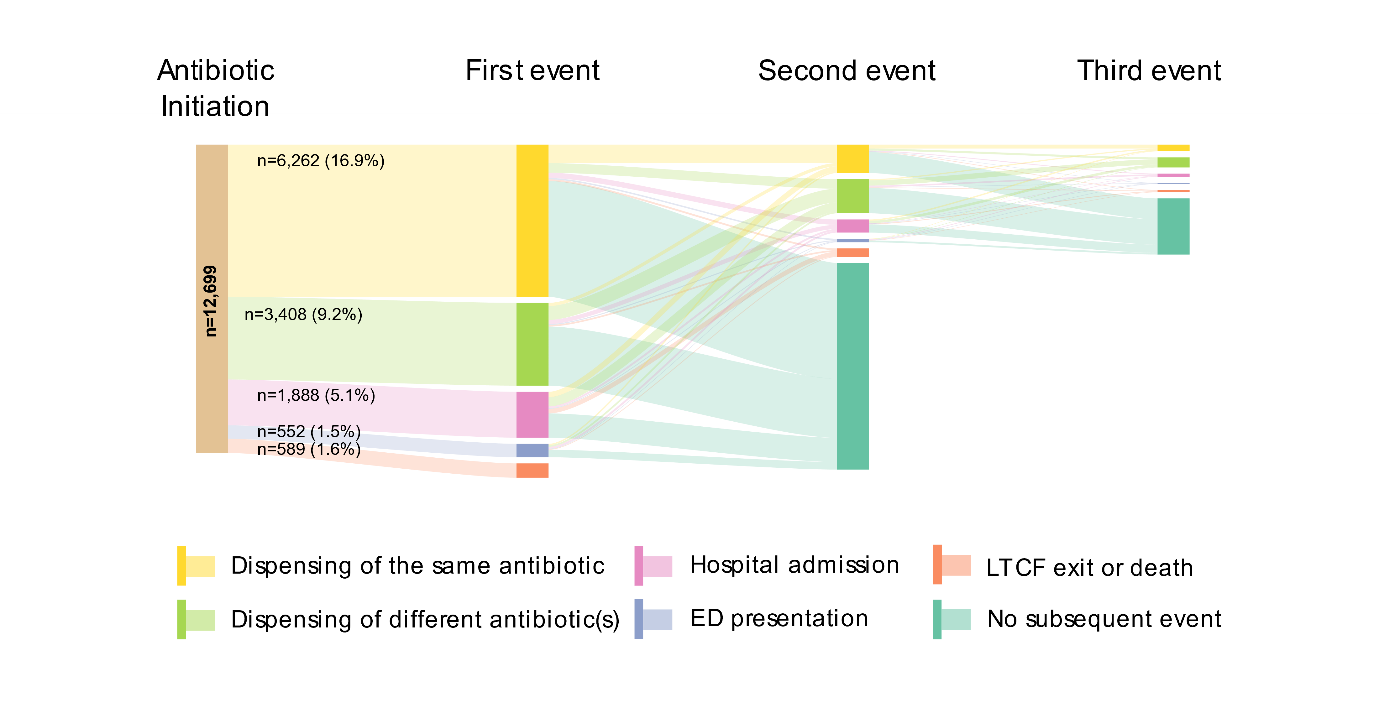


ED, emergency department.

Individuals who did not experience an event in the 14-day follow-up period are not shown. Secondary outcomes of interest experienced after the third event and within 14 days were examined but are not displayed in the figure due to low prevalence (n=256, 2.0% of people with ≥1 event). The width of pathways plotted for 1-5 individuals were determined as the width of three individuals for statistical disclosure control. If >1 antibiotic was dispensed on the date of study entry and another antibiotic was supplied in the 14-day follow-up period the resident was considered to have received the ‘same antibiotic’ if it was the same as at least one of those supplied at study entry, or a ‘different antibiotic’ if it was different to all of the antibiotics supplied initially.

**Figure S4.** Adjusted odds ratio with 95% confidence intervals for logistic regression models evaluating resident characteristics associated with provision of a urine test around the time of antibiotic initiation.

**
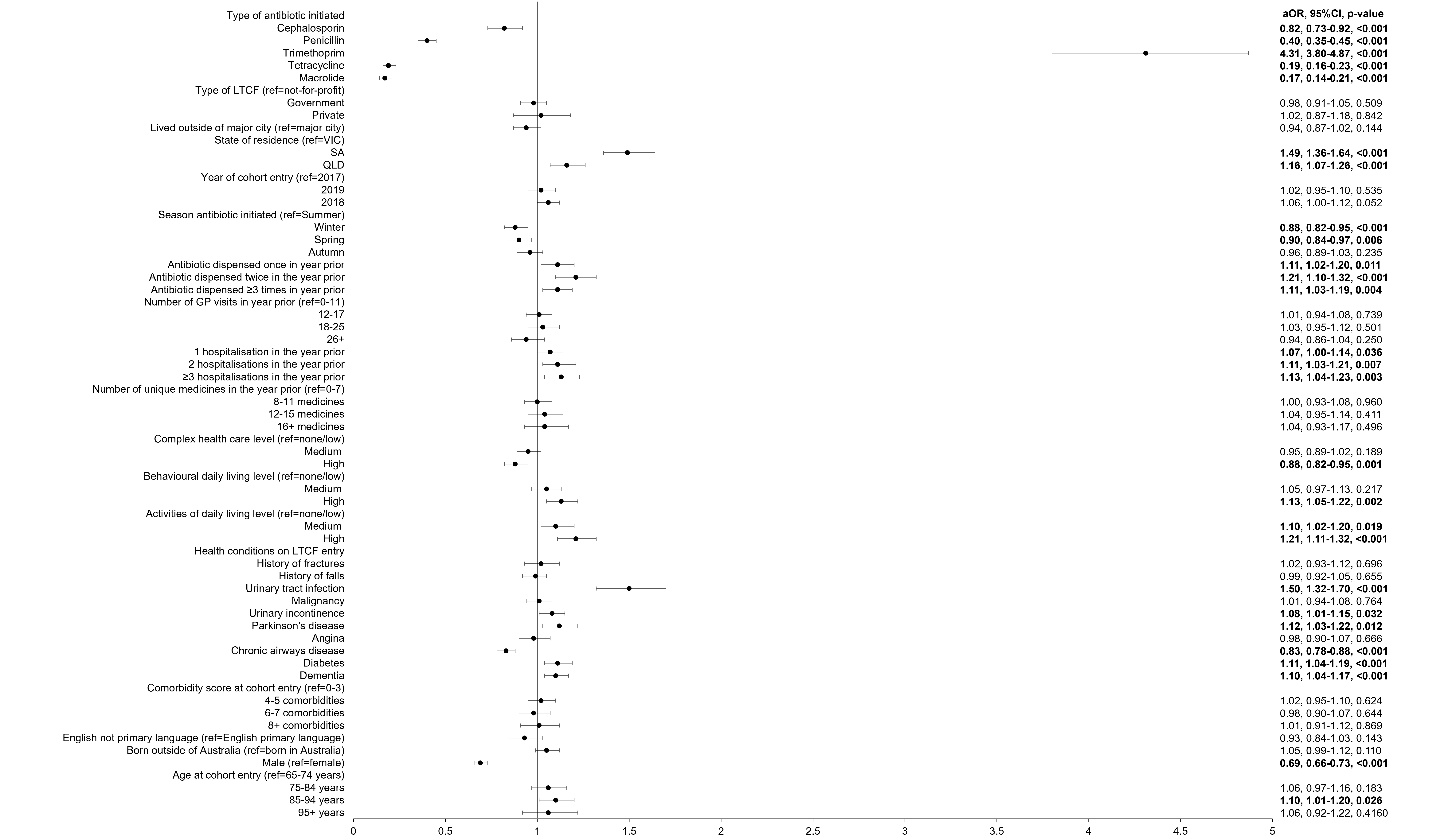
**

aOR, adjusted odds ratio, CI, confidence interval, LTCF, long-term care facility, SA, South Australia, QLD, Queensland, VIC, Victoria. There were n=611 (1.7%) residents with missing data who were excluded for complete case analysis. Nitrofurantoin, lincosamide, quinolones, nitroimidazole, aminoglycosides, glycopeptides and other systemic antibiotics were not included in the model due to low prevalence of use (each class <1.5% of antibiotics dispensed). Statistically significant results shown in bold.
